# Meta-analysis of Glutamine on Immune Function and Post-Operative Complications of Patients With Colorectal Cancer

**DOI:** 10.3389/fnut.2021.765809

**Published:** 2021-12-06

**Authors:** Tao Yang, Xuhong Yan, Yibo Cao, Tiantian Bao, Guangsong Li, Shengliang Gu, Kai Xiong, Tianbao Xiao

**Affiliations:** ^1^Colorectal and Anal Surgery, The First Affiliated Hospital of Guizhou University of Traditional Chinese Medicine, Guiyang, China; ^2^Department of Dermatovenereology, Chengdu Second People's Hospital, Chengdu, China; ^3^Department of Pharmacy, The First Affiliated Hospital of Guizhou University of Traditional Chinese Medicine, Guiyang, China; ^4^College of Clinical Medicine, Guizhou University of Traditional Chinese Medicine, Guiyang, China

**Keywords:** colorectal cancer, humoral immunity, T cell immunity, post-operative complications, meta-analysis

## Abstract

The aim of this meta-analysis was to evaluate the clinical significance of glutamine in the management of patients with colorectal cancer (CRC) after radical operation. Electronic databases, including PubMed, EMBASE, MEDLINE, Cochrane Library, Chinese Biomedical Database (CBM), China National Knowledge Infrastructure (CNKI), VIP medicine information system (VIP), and Wanfang electronic databases were comprehensively searched from inception to 30, July 2021. Prospective randomized trials with glutamine vs. routine nutrition or blank therapy were selected. The immune function related indicators (including IgA, IgG, IgM, CD4+, CD8+, and the ratio of CD4+/CD8+), post-operative complications [including surgical site infection (SSI), anastomotic leakage, and length of hospital stay (LOS)], and corresponding 95% confidence intervals (CIs) were assessed in the pooled analysis. Subsequently, the heterogeneity between studies, sensitivity, publication bias, and meta-regression analysis were performed. Consequently, 31 studies which contained 2,201 patients (1,108 in the glutamine group and 1,093 in the control group) were included. Results of pooled analysis indicated that glutamine significantly improved the humoral immune function indicators [including IgA (SMD = 1.15, 95% CI: 0.72–1.58), IgM (SMD = 0.68, 95% CI: 0.48–0.89), and IgG (SMD = 1.10, 95% CI: 0.70–1.50)], and the T cell immune function indicators [including CD4+ (SMD = 0.76, 95% CI: 0.53–0.99) and the ratio of CD4+/CD8+ (SMD = 0.92, 95% CI: 0.57–1.28)]. Meanwhile, the content of CD8+ was decreased significantly (SMD = −0.50, 95% CI: −0.91 to −0.10) followed by glutamine intervention. Pooled analysis of SSI (RR = 0.48, 95% CI: 0.30–0.75), anastomotic leakage (RR = 0.23, 95% CI: 0.09–0.61), and LOS (SMD = −1.13, 95% CI: −1.68 to −0.58) were decreased significantly in glutamine group compared with control group. Metaregression analysis revealed that the covariate of small-sample effects influenced the robustness and reliability of IgG outcome potentially. Findings of the present work demonstrated that glutamine ought to be applied as an effective immunenutrition therapy in the treatment of patients with CRC after radical surgery. The present meta-analysis has been registered in PROSPERO (no. CRD42021243327).

**Systematic Review Registration:** https://www.crd.york.ac.uk/PROSPERO, Identifier: CRD42021243327.

## Introduction

Colorectal cancer (CRC) is the most malignant tumors in digestive system and has become a serious threat to human health. Statistically, the global data showed that newly increased patients with CRC ~1,880,725 (including 1,148,515 cases of colon cancer and 732,210 cases of rectum cancer), and the fatality rate of CRC was estimated to be ~9.4% (1). Furthermore, the death rate from CRC is predicted to increase by 60% (colon cancer) and 71.5% (rectum cancer), respectively, in 2035 (2). Data from the American Cancer Society indicates that CRC is the third most common cancer diagnosed in both men and women in the United States. The number of CRC cases in the US for 2021 are: 1,04,270 new cases of colon cancer and 45,230 new cases of rectal cancer (3). From 2012 through 2016, CRC increased every year by 2% in people younger than 50 and 1% in people 50–64 in the US (3).

Surgical treatment in the management of non-metastatic or resectable CRC is irreplaceable and recommended as the first-line for radical treatment by the National Comprehensive Cancer Network (NCCN) Guidelines (4, 5) and European Society for Medical Oncology (ESMO) Guidelines (6, 7). However, due to the long-term consumption of tumor before the radical resection of CRC, insufficient nutritional intake, and the stress responses caused by surgical trauma the patients are most likely to suffer from malnutrition, decreased immune function, intestinal dysfunction, and post-operative complications. Previous studies have reported that malnutrition prevalence has been widely reported to reach 15–40% in patients with cancer at the time of diagnosis, and up to 80–90% in advanced cases of the disease (8). The prevalence of malnutrition in CRC patients also ranged from 45 to 60% (9) and these rates significantly increased followed by radical surgery (10). In addition, immune dysfunction or immunosuppression caused by surgery acted as the main inducement of post-operative complications. Many studies have attributed post-operative complications such as surgical site infection (SSI), anastomotic leak, ureteral injury, intraabdominal abscess, enteric fistula, bleeding, and post-operative bowel obstruction to immune dysfunction and malnutrition (11–14). Consequently, these complications not only significantly affected the short-term outcomes, such as the prolonging length of hospital stay (LOS) and increasing associated health costs, but it also deteriorated the long-term oncological results, including declining patients' quality of life and cancer recrudescence (15, 16).

Increasing evidences from clinical researches demonstrated that immunonutrition therapy was very likely to improve the immune function and decrease complications or recrudescence in patients after CRC surgery (17–20). Glutamine, a critical substance of immunenutrition, is an important source of energy for the intestinal tract and could improve intestinal function. Many studies have revealed the positive role of glutamine in CRC patients who underwent radical surgery (21–23). Furthermore, glutamine levels in serum could affect overall survival (OS) and progression-free survival (PFS) significantly, and serum glutamine levels may be applied as a prognostic indicator in patients with CRC (24, 25). However, other studies indicated that glutamine applied in CRC patients did not significantly improve the survival outcomes or post-operative complications (26–28).

These evidences were hard to match due to the heterogeneity of study designs, study populations, sample quantities, and systematic approaches based upon current clinical studies. To address those ambiguities and to evaluate the actual clinical significance of glutamine in patients with CRC, a meta-analysis of randomized, prospective clinical trials about glutamine applied in CRC patients who underwent radical surgery was conducted. This meta-analysis provided essential evidence of the effects of glutamine on immune functions and post-operative complications of patients with CRC.

## Materials and Methods

### Protocol Registration

We have registered this protocol previously in PROSPERO in April 2021 (number: CRD42021243327, https://www.crd.york.ac.uk/PROSPERO).

### Eligibility Criteria

The Cochrane Handbook for Systematic Reviews of Interventions and the PRISMA statement was referred by this study and the “PICOS” principles was employed for developing the inclusion and exclusion criteria. Studies that meet the following inclusion criteria were included: (1) the design of study was a prospectively randomized controlled trial (RCT); (2) patients with CRC (including colon cancer and rectal cancer) and undergone radical surgery; (3) glutamine was set as experiment group and routine nutrition or blank therapy (fluid supporting therapy) as control group; (4) at least one of the investigated outcomes was reported in original researches. The exclusion criteria were as follows: (i) irrelevant studies and duplicated literatures; (ii) unavailable data literatures; (iii) letters, reviews, comments, case-report, laboratory studies, and meta-analysis.

### Search Methodology

The PubMed, EMBASE, MEDLINE, Cochrane Library, Chinese Biomedical Database (CBM), China National Knowledge Infrastructure (CNKI), VIP medicine information system (VIP), and Wanfang electronic databases were comprehensively searched up to July 30, 2021. The search terms were in the combination of medical subject headings (MeSH) terms and the following free words: (Colon/Rectal/colorectal/cancer/tumor/carcinoma/neoplasm) AND (glutamine/nutrition/immunenutrition) AND (complication/infection/leakage) AND (immune/immunity/IgA/IgG/IgM/CD4+/CD8+/CD4+/CD8+) AND (random/randomized/RCTs/clinical trial). In addition, potentially relevant references were also obtained manually. The language of all the publications was not limited.

### Study Selection

All search results were combined in Endnote™, Version X8 (Thompson Reuters). Duplicates were removed manually. Two investigators (Tao Yang and Xuhong Yan) filtered the original studies independently. If the literature meets the eligibility criteria, the two investigators will further read the full text to screen the study. Any discrepancies were tackled by discussion or third-party consensus.

### Data Extraction and Analysis

All data were collected independently by two investigators (Tao Yang and Yibo Cao) from eligible RCTs using a standardized form. The following information were extracted including: (i) Study ID, including the name of the first author and publication year; (ii) study subjects, number of participants, and their ages; (iii) treatment regimens for the treatment and control groups; and (iv) the primary endpoint, the immune function related indicators (including IgA, IgG, IgM, CD4+, CD8+, and the ratio of CD4+/CD8+) and the secondary endpoint, the post-operative complications (including SSI, anastomotic leakage, and LOS). If insufficient details were reported, we would contact authors for further information. Disagreements between two investigators were tackled by discussion and consensus.

### Quality Assessment

The Cochrane Collaboration's tool for assessing risk of bias were employed for quality evaluation. Any disagreements during assessment were resolved by iteration, discussion, and consensus.

### Statistical Analysis

All data were analyzed using Stata version 14.0 (Stata Corporation). Heterogeneity amongst studies was assessed using a *Q*-test and an *I*^2^-test before determining the pooled effect. A fixed effects model or a random effects model was based on the results of the *Q*-test and *I*^2^-test. A fixed effects model was adopted if *I*^2^ < 50% and *p* > 0.1. Otherwise, a random effects model was used. The primary endpoint was immune function related indicators (IgA, IgG, IgM, CD4^+^, CD8^+^, and CD4^+^/CD8^+^) and LOS was a continuous variable. The pooled analysis of these indicators was expressed as standard mean difference (SMD). The SSI and anastomotic leakage were dichotomous variables, and the pooled analysis of these complications was expressed as relative risks (RR). The significance of pooled effects was determined using a *Z*-test; *p* < 0.05 was considered to indicate a statistically significant difference. Sensitivity analysis was utilized to investigate the influence of a high-risk study on overall meta-analysis. Possible publication bias and the detailed reasons underlying publication bias were determined by contour-enhanced funnel plot. Possible source of heterogeneity was explored by metaregression performing via random effect model, and the restricted maximum likelihood (REML) estimation method proposed by Harbord et al. (29) was applied in metaregression.

## Results

### Study Selection Outcome

**Figure 1:**
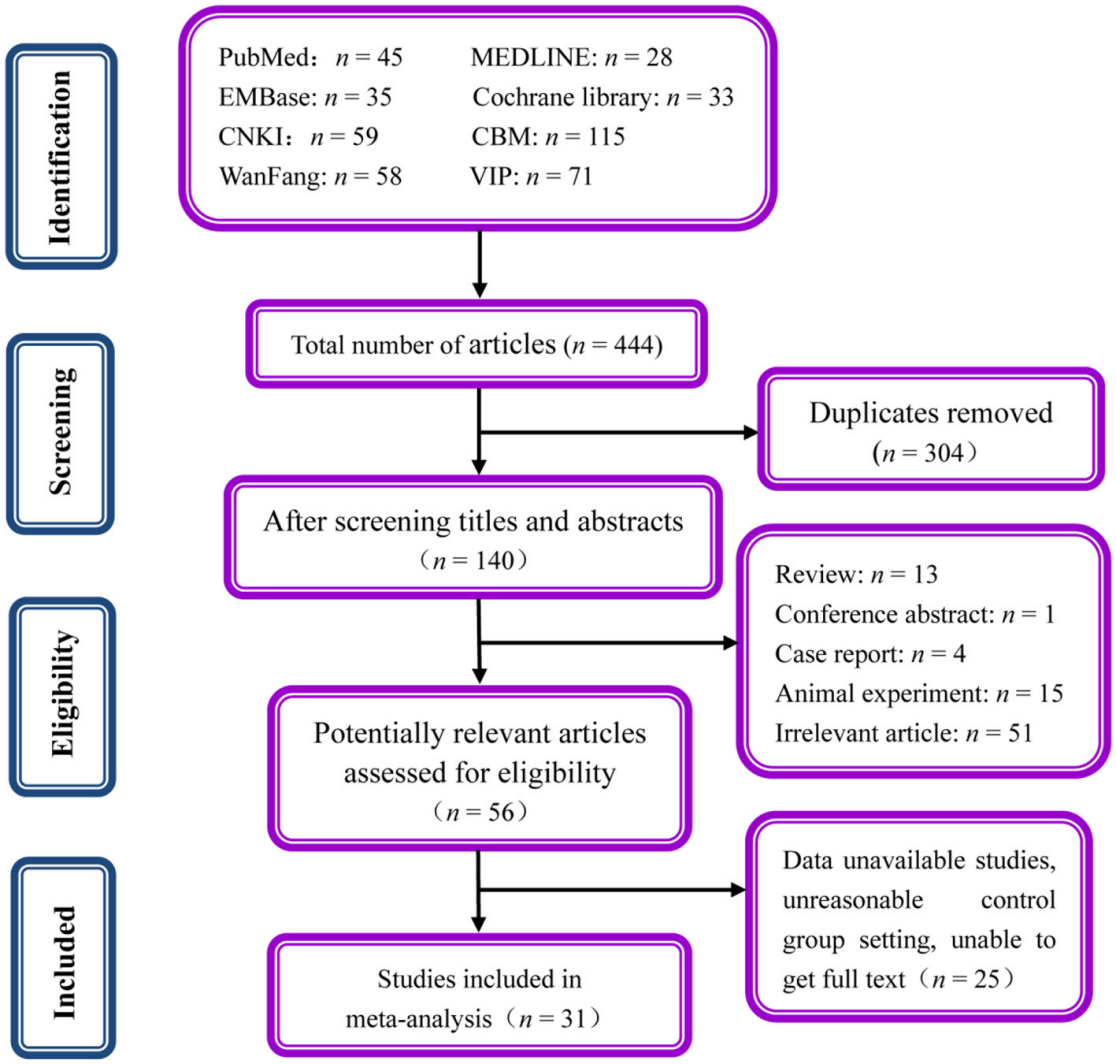
Flowchart presenting the selection process of studies.

A total of 444 relevant articles were retrieved ultimately. Among these, 304 were repeated articles. Totally, 84 literatures were excluded by screening the titles and abstracts due to reviews, conference abstract, animal experiments, case report, with 56 articles remaining. Then 25 articles were excluded by examining the abstracts or full-texts. Finally, this meta-analysis included 31 studies that fulfilled the inclusion criteria (Figure 1).

### Study Characteristics

Totally, 2,201 patients were involved in 31 studies (21–23, 30–57). Among these, 1,108 were allocated to the glutamine group and 1,093 patients were allocated to the control group. Table 1 displayed the main characteristics of the included 31 studies. Overall, 31 studies were published between 1998 and 2019 years. Eight trials (22, 30, 31, 34, 38, 45, 46, 57) administrated glutamine through enteral nutrition (EN) and 23 trials (21, 23, 32, 33, 35–37, 39–44, 47–56) administrated through parenteral nutrition (PN). With regards to the outcomes of humoral immune function, 14 trials (31, 32, 37, 38, 41–44, 47–50, 54, 56) reported IgA indicator, 17 trials (31, 32, 34, 35, 37, 38, 41–44, 47–50, 52, 54, 56) reported IgM indicator, and 17 trials (31, 32, 34, 35, 37, 38, 41–44, 47–50, 52, 54, 56) reported IgG indicator. In addition, the outcomes of T cell immune function, including CD4+ content, was reported by 16 trials (30, 31, 33, 34, 37–40, 43, 44, 47, 49, 50, 52, 56, 57), CD8+ content, was reported by 15 trials (30, 33, 34, 37–40, 43, 44, 47, 49, 50, 52, 56, 57), and the ratio of CD4+/CD8+ was reported by 13 trials (31, 33, 34, 37, 38, 40, 44, 47, 49, 50, 52, 56, 57). Furthermore, the outcome of anastomotic leakage was reported by seven trials (22, 23, 35, 39, 41, 45, 51), SSI was reported by 12 trials (22, 23, 32, 35, 36, 39–41, 45, 46, 51, 53), and the LOS was reported by eight trials (21, 23, 36, 38, 45, 46, 48, 55). The main characteristics of the included studies are presented in Table 1.

**Table 1:** Main information of included studies in the meta-analysis.

**Study ID**	**Sample size (** ***n*** **)**		**Ages (year)**		**Dose of glutamine**	**Route of administration**	**Tumor types**	**Outcomes**
	**Treatment**	**Control**	**Treatment**	**Control**				
Morlion et al. (21)	15	13	Mean: 67.1	Mean: 68.2	0.3 g/(kg•d)	PN	CRC	⑨
Oguz et al. (23)	57	52	Mean: 52	Mean: 57	1 g/(kg•d)	PN	CRC	⑦ ⑧ ⑨
Cui et al. (55)	20	20	Mean: 55	Mean: 56	0.5 g/(kg•d)	PN	CC	⑨
van Barneveld et al. (22)	61	62	Mean: 64	Mean: 65	11.9 g/d	EN	RC	⑦ ⑧
Chen (38)	22	22	58.7 ± 6.7		30 g/d	EN	CRC	① ② ③ ④ ⑤ ⑥ ⑨
Chen et al. (31)	50	50	64.22 ± 5.89	63.57 ± 6.5	60 g/d	EN	RC	① ② ③ ④ ⑥
Chen and Lin (39)	24	24	66.84 ± 5.52	68.12 ± 4.46	0.4 g/(kg•d)	PN	CC	④ ⑤ ⑥ ⑦ ⑧
Chen et al. (40)	42	42	62.1 ± 10.6	62.7 ± 11.3	0.5 g/kg•d	PN	CC	④ ⑤ ⑥ ⑧
Cheng and Huang (41)	50	50	NR		100 ml/d	PN	CC	① ② ③ ⑦ ⑧
De et al. (57)	52	52	53.54 ± 11.57	53.24 ± 11.38	100 ml/d	EN	CC	④ ⑤ ⑥
Huang et al. (42)	63	63	Range: 32–69	Range: 35–67	100 ml/d	PN	CC	① ② ③
Huang et al. (43)	15	15	57.0 ± 4.7	56.8 ± 3.5	0.4 g/(kg•d)	PN	CRC	② ③ ④ ⑤
Huang et al. (35)	11	11	Range: 41–70		100 ml/d	PN	CRC	① ② ③ ⑦ ⑧
Jiang et al. (44)	31	31	56.8 ± 10.2	58.2 ± 9.5	0.4 g/(kg•d)	PN	CRC	① ② ③ ④ ⑤ ⑥
Li et al. (36)	20	20	57.81 ± 3.75	58.02 ± 4.63	NR	PN	CRC	⑧ ⑨
Li and Jia (45)	32	32	62.6 ± 9.6	65.5 ± 9.0	0.5 g/(kg•d)	EN	CRC	⑦ ⑧ ⑨
Li and Li (30)	30	30	50.1 ± 4.6	50.5 ± 4.9	0.4 g/(kg•d)	EN	RC	④ ⑤
Liu et al. (46)	40	40	61.4 ± 7.0	59.1 ± 7.5	100 ml/d	EN	CC	⑧ ⑨
Liu et al. (47)	43	42	57.1 ± 9.8	58.2 ± 10.1	0.4 g/(kg•d)	PN	CRC	① ② ③ ④ ⑤ ⑥
Luo et al. (48)	23	23	Range: 38–69		0.5 g/(kg•d)	PN	CC	① ② ③ ⑨
Shao et al. (34)	51	51	Range: 35–75		NR	EN	CRC	② ③ ④ ⑤ ⑥
Song et al. (49)	20	20	Range: 28–80		0.4 g/(kg•d)	PN	CRC	① ② ③ ④ ⑤ ⑥
Ya et al. (50)	24	24	NR		20 g/d	PN	CRC	① ② ③ ④ ⑤ ⑥
Wang et al. (32)	30	30	58.7 ± 3.6	60.3 ± 4.5	0.3 g/(kg•d)	PN	RC	① ② ③ ⑧
Yang and Li (51)	24	20	Mean: 60.2	Mean: 61.1	100 mL/d	PN	CC	⑦ ⑧
Tasheng et al. (33)	70	70	59.3 ± 8.2	55.3 ± 9.1	0.4 g/(kg•d)	PN	CRC	④ ⑤ ⑥
Zhang et al. (37)	47	47	57.35 ± 16.4		100 mL/d	PN	CRC	① ② ③ ④ ⑤ ⑥
Zhang and Li (52)	30	30	Range: 28–80		0.4 g/(kg•d)	PN	CC	② ③ ④ ⑤ ⑥
Zhao (53)	32	28	56.75 ± 5.60	54.42 ± 5.21	50 mL/d	PN	CRC	⑧
Zheng (54)	55	55	NR		100 mL/d	PN	CC	① ② ③
Bu et al. (56)	24	24	70.5 ± 10.6	66.8 ± 10.9	0.5 g/kg•d	PN	CRC	① ② ③ ④ ⑤ ⑥

*NR, not report; PN, parenteral nutrition; EN, enteral nutrition; ①, CD4+; ②, CD8+; ③, the ratio of CD4+/CD8+; ④, IgA; ⑤, IgG; ⑥, IgM; ⑦, anastomotic leakage; ⑧, SSI; ⑨, LOS*.

### Study Quality Assessment

**Figure 2:**
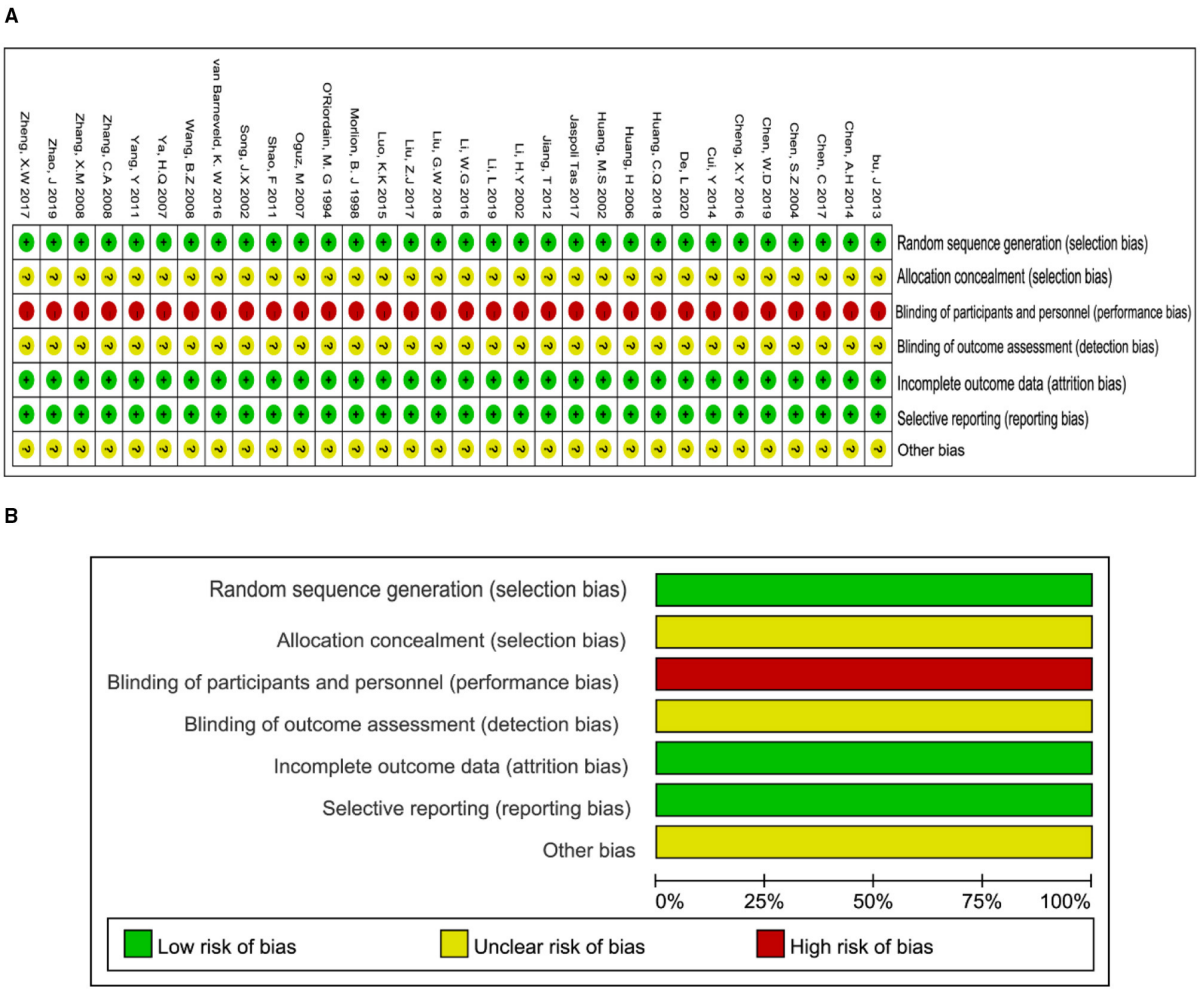
Methodological quality graph and summary of the included studies: **(A)** Risk of bias summary; **(B)** Risk of bias graph.

Methodological quality assessment and outline of the included 31 studies were presented in Figures 2A,B. The generation of randomized sequence was identified adequately in all trials. The allocation concealment was unclear according to all trials. These trials were neither single nor double blinding design. Consequently, the evaluation of detection bias was high risk (Figure 2B). Incomplete outcomes and selective reporting were not detected in all studies. Conclusively, the methodological quality of all included trials stayed at a lower level due to the lack of blinding.

### Results of Meta-analysis

#### Glutamine on Humoral Immune Function of Patients With CRC

**Figure 3:**
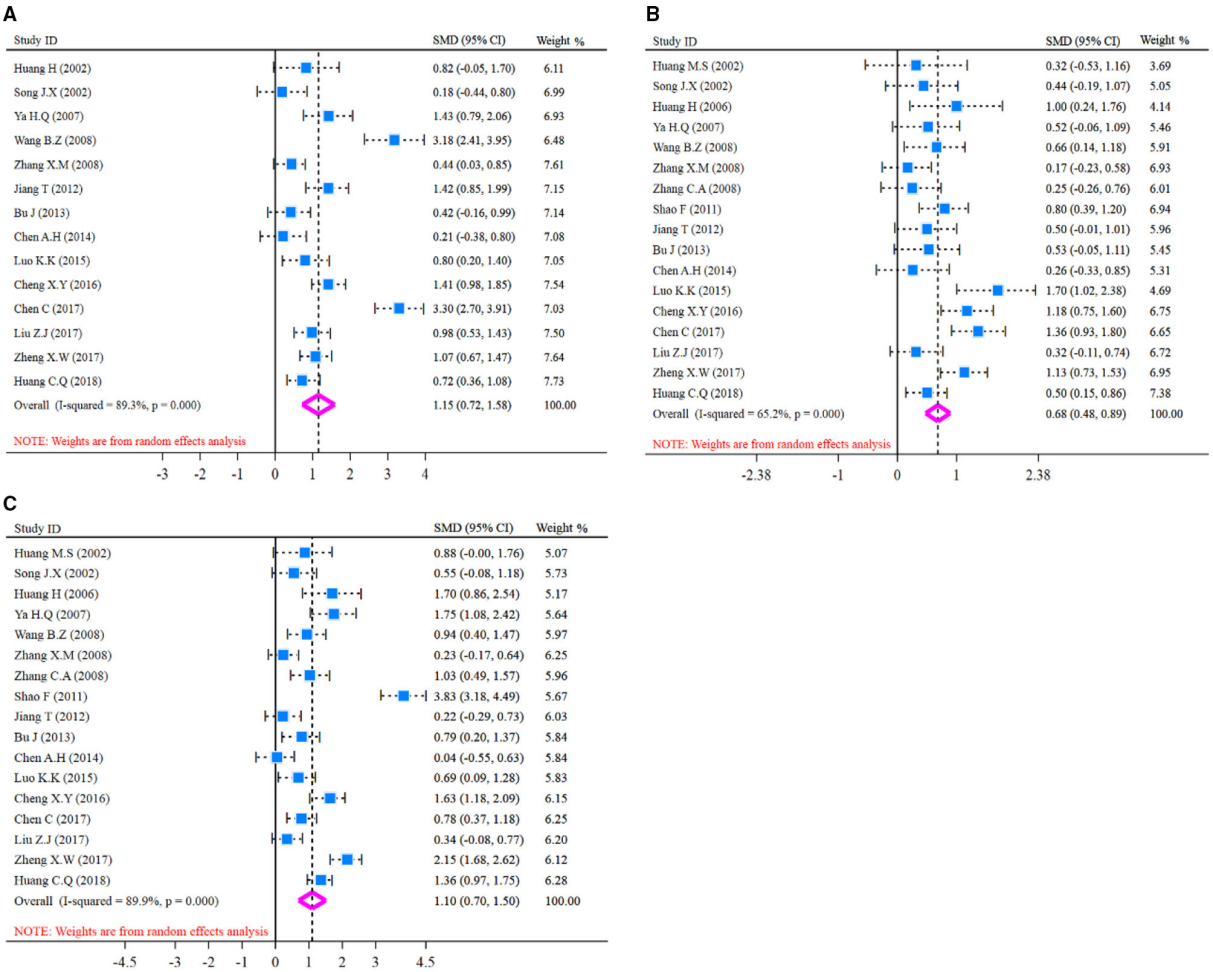
Forest plot of standard mean difference (SMD) for IgA, IgM, and IgG. **(A)** Forest plot of IgA; **(B)** Forest plot of IgM; **(C)** Forest plot of IgG. All pooled analysis applied a random effect model.

The pooled analysis of humoral immune function indicators (IgA, IgM, IgG) is presented in Figure 3 and SMD presentation. Heterogeneity was examined firstly before pooled analysis of these indicators. Test results revealed that there was a significant heterogeneity for IgA (*I*^*2*^-test = 89.3% and *Q-*test *p* = 0.000, Figure 3A), moderate heterogeneity for IgM (*I*^*2*^-test = 65.2% and *Q-*test *p* = 0.000, Figure 3B), and significant heterogeneity for IgG (*I*^*2*^-test = 89.9% and *Q-*test *p* = 0.000, Figure 3C) between included studies. Thus, a random effect model was selected for pooled analysis. Results revealed that IgA content was significantly increased (*Z* = 5.27, *p* = 0.000; SMD = 1.15, 95% CI: 0.72–1.58; Figure 3A) in the glutamine group compared with the control group. Meanwhile, the indicator of IgM was also increased (*Z* = 6.47, *p* = 0.000; SMD = 0.68, 95% CI: 0.48–0.89; Figure 3B) in glutamine group. In addition, the indicator of IgG was significantly increased (*Z* = 5.34, *p* = 0.000; SMD = 1.10, 95% CI: 0.70–1.50; Figure 3C) in glutamine group compared with control group. These results demonstrated that glutamine improved the humoral immune function effectively for patients with CRC after radical operation.

#### Glutamine on T Cell Immune Function of Patients With CRC

**Figure 4:**
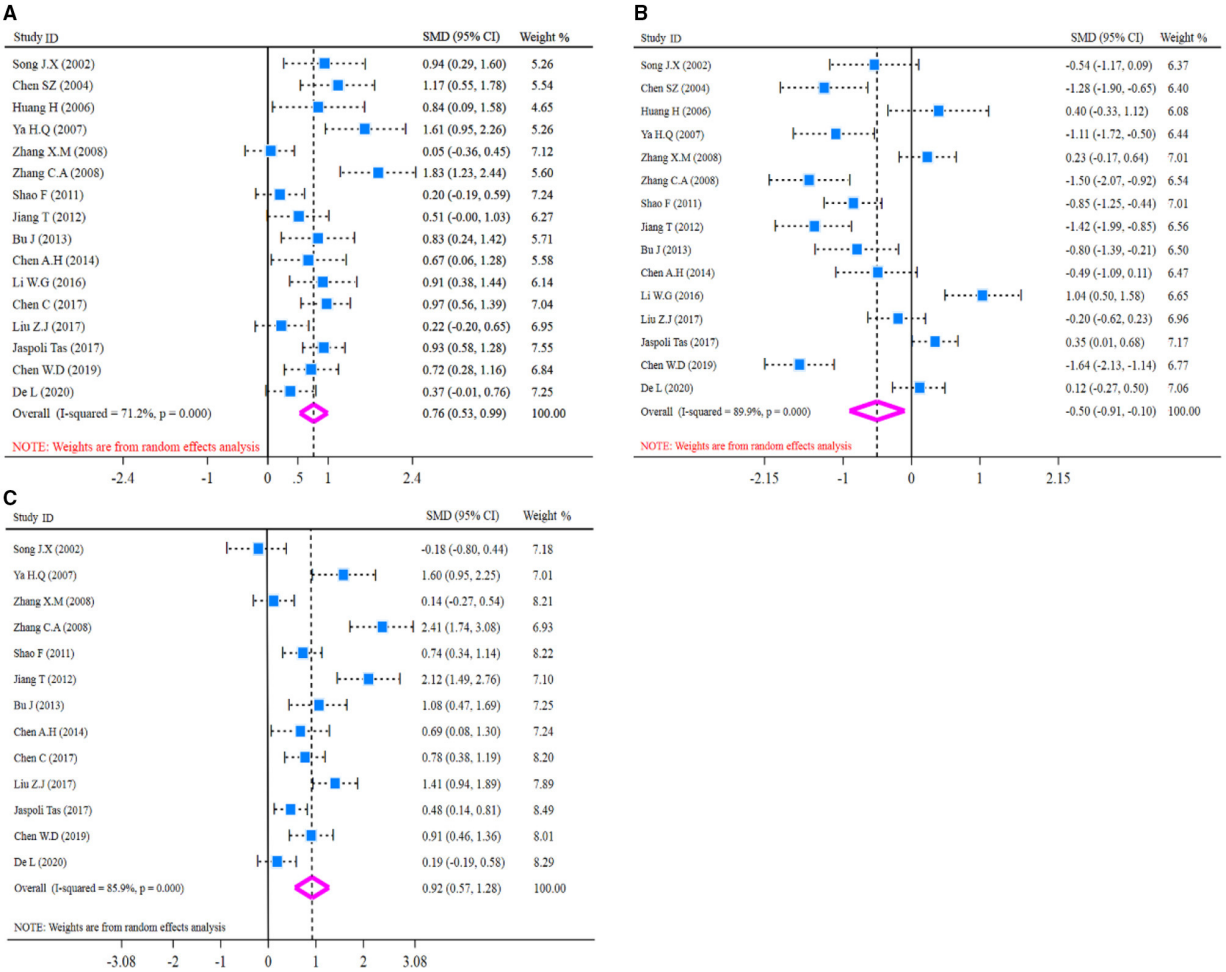
Forest plot of standard mean difference (SMD) for CD4+, CD8+, and CD4+/CD8+. **(A)** Forest plot of CD4+; **(B)** Forest plot of CD8+; **(C)** Forest plot of CD4+/CD8+. All pooled analysis applied a random effect model.

Before pooled analysis of T cell immune function indicators (CD4+, CD8+, CD4+/CD8+), heterogeneity across studies was tested conventionally. Heterogeneity test results revealed there was moderate heterogeneity for CD4+ (*I*^*2*^-test = 71.2% and *Q-*test *p* = 0.000, Figure 4A), significant heterogeneity for CD8+ (*I*^*2*^-test = 89.9% and *Q-*test *p* = 0.000, Figure 4B), and significant heterogeneity for CD4+/CD8+ (*I*^*2*^-test = 85.9% and *Q-*test *p* = 0.000, Figure 4C). So, a random effect model was selected for pooled analysis. In the pooled meta-analysis, the content of CD4+ was increased significantly (*Z* = 6.47, *p* = 0.000; SMD = 0.76, 95% CI: 0.53–0.99; Figure 4A) in the glutamine group compared with the control group. On the contrary, the content of CD8+ was decreased significantly (*Z* = 2.44, *p* = 0.015; SMD = −0.50, 95% CI: −0.91 to −0.10; Figure 4B) in the glutamine group. Meanwhile, the ratio of CD4+/CD8+ was increased significantly (*Z* = 5.07, *p* = 0.000; SMD = 0.92, 95% CI: 0.57–1.28; Figure 4C) in the glutamine group compared with the control group. Results are shown in Figure 4 and SMD presentation.

#### Glutamine on Post-Operative Complications of Patients With CRC

**Figure 5:**
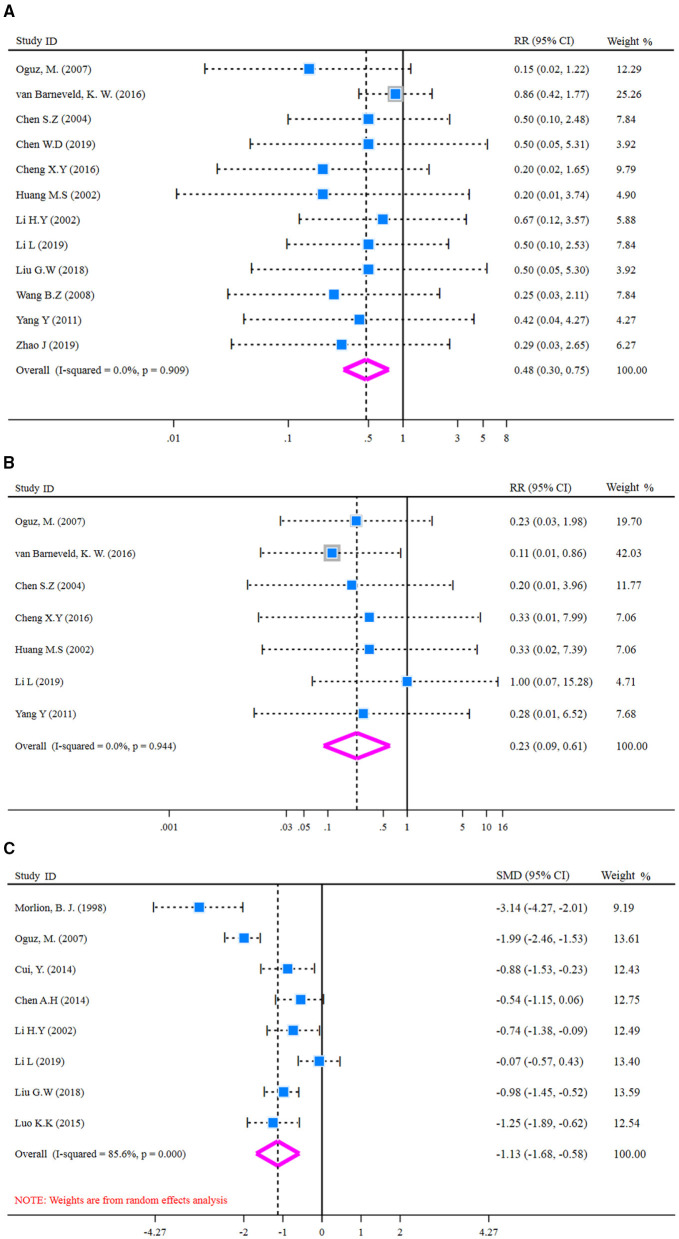
Forest plot of for SSI, anastomotic leakage, and LOS. **(A)** Forest plot of SSI applied a fixed effect model; **(B)** Forest plot of anastomotic leakage applied a fixed effect model; **(C)** Forest plot of LOS applied a random effect model. SSI, surgical site infection; LOS, length of hospital stay.

Heterogeneity was examined prior to pooled analysis of SSI, anastomotic leakage, and LOS. Test results revealed there were no significant heterogeneity across 12 studies (*I*^*2*^-test = 0.0% and *Q-*test *p* = 0.909, Figure 5A) that reported SSI outcome, seven studies (*I*^*2*^-test = 0.0% and *Q-*test *p* = 0.944, Figure 5B) that reported anastomotic leakage. Thus, a fixed effects model was applied for the pooled analysis. However, results revealed there was significant heterogeneity for LOS outcome (*I*^*2*^-test = 85.6% and *Q-*test *p* = 0.000, Figure 5C). So, a random effect model was employed for pooled analysis. In the pooled meta-analysis, the rates of SSI were decreased significantly (*Z* = 3.18, *p* = 0.001; RR = 0.48, 95% CI: 0.30–0.75; Figure 5A) in glutamine group compared with the control group. Meanwhile, the rates of anastomotic leakage were decreased significantly (*Z* = 2.98, *p* = 0.003; RR = 0.23, 95% CI: 0.09–0.61; Figure 5B) in the glutamine group. Furthermore, the LOS outcome was decreased significantly (*Z* = 4.03, *p* = 0.000; SMD = −1.13, 95% CI: −1.68 to −0.58; Figure 5C) in the glutamine group compared with the control group. These results showed that glutamine could reduce post-operative complications of patients with CRC.

#### Sensitivity Analysis for Robustness of Pooled Analysis

**Figure 6:**
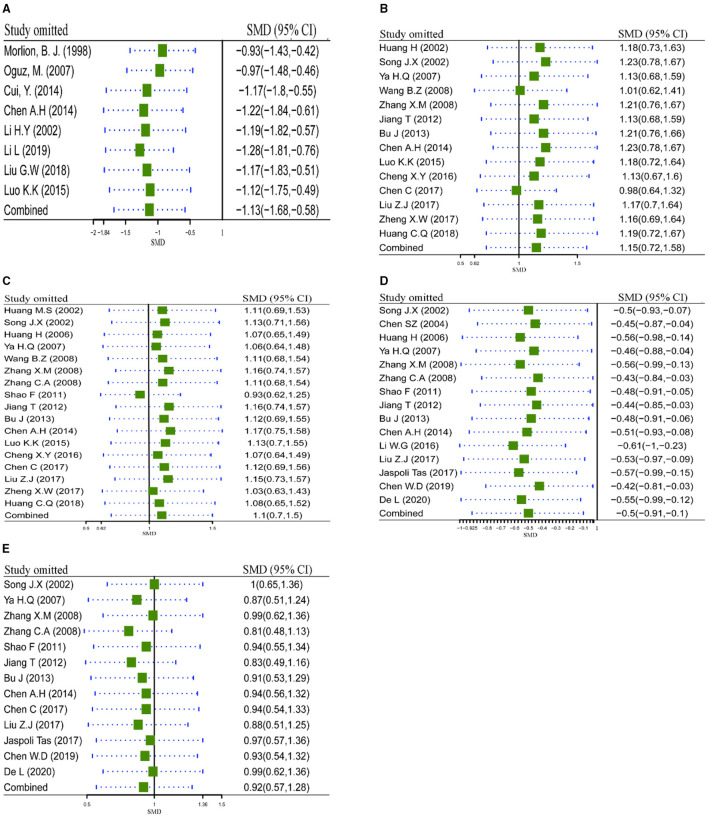
Sensitivity analysis via leave-one-out procedure each time. **(A)** Sensitivity analysis of LOS; **(B)** Sensitivity analysis of IgA; **(C)** Sensitivity analysis of IgG; **(D)** Sensitivity analysis of CD8+; **(E)** Sensitivity analysis of CD4+/CD8+. LOS, length of hospital stay.

Sensitivity analysis *via* leave-one-out procedure each time was carried out to verify robustness of pooled results (LOS, IgA, IgG, CD8+, and CD4+/CD8+) with significant heterogeneity (≥80%) across included studies. Results are shown in Figure 6. Sensitivity analysis of LOS outcome (Figure 6A) indicated that exclusion of any study did not account for heterogeneity significantly, which demonstrated the pooled result of LOS was robust to some extent. Meanwhile, the same conclusions were retrieved from the sensitivity analysis of IgA (Figure 6B), IgG (Figure 6C), CD8+ (Figure 6D), and CD4+/CD8+ (Figure 6E). All results of sensitivity analysis demonstrated that the pooled results were robust to some extent.

#### Contour-Enhanced Funnel Plot for Potential Source of Publication Bias

**Figure 7:**
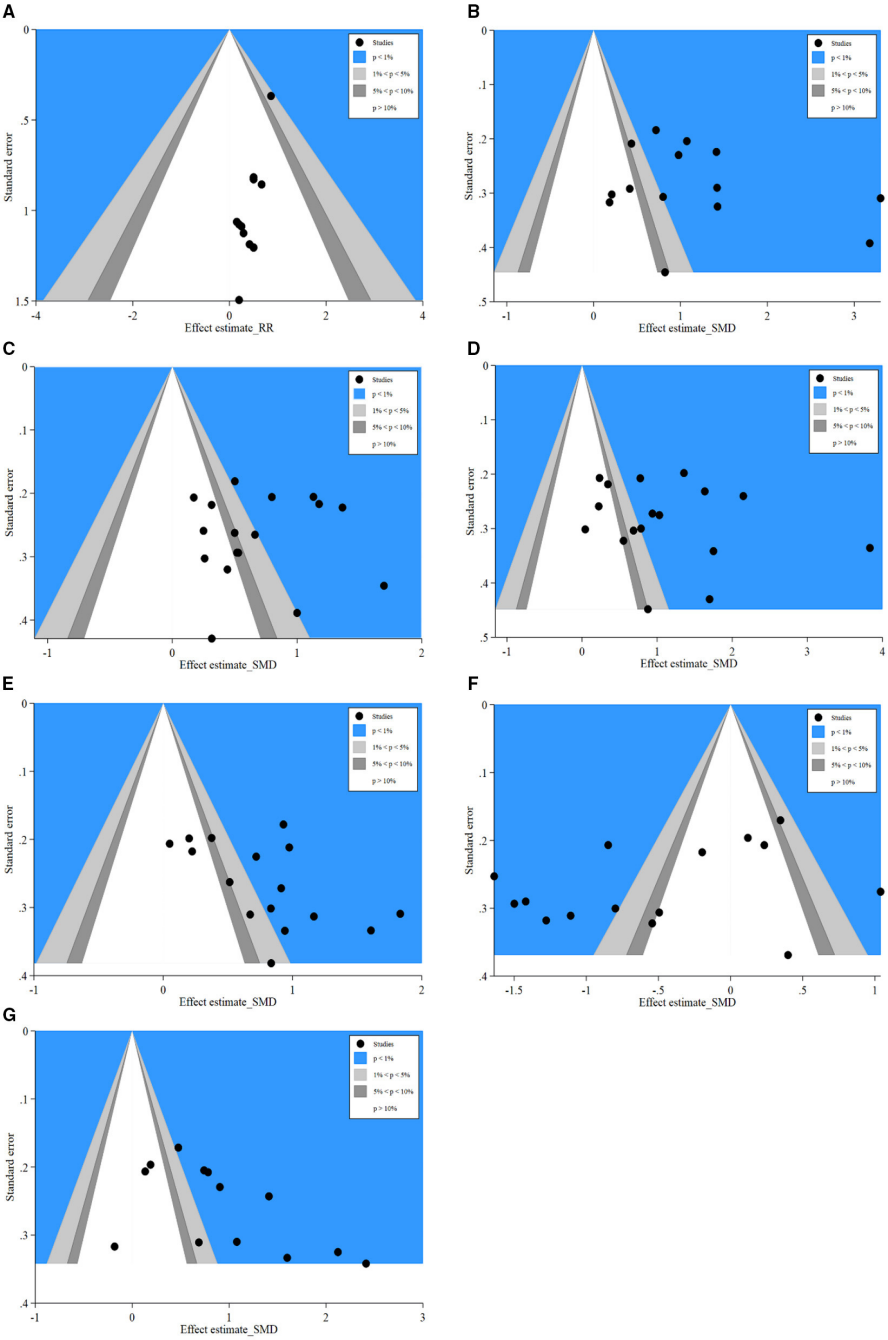
Contour-enhanced funnel plots of SSI, IgA, IgM, IgG, CD4+, CD8+, and CD4+/CD8+. **(A)** Contour-enhanced funnel plot of SSI; **(B)** Contour-enhanced funnel plot of IgA; **(C)** Contour-enhanced funnel plot of IgM; **(D)** Contour-enhanced funnel plot of IgG; **(E)** Contour-enhanced funnel plot of CD4+; **(F)** Contour-enhanced funnel plot of CD8+; **(G)** Contour-enhanced funnel plot of CD4+/CD8+. SSI, surgical site infection.

Contour-enhanced funnel plot, which added conventional milestones in levels of statistical significance (*p* < 0.01, *p* < 0.05, *p* < 0.1 or *p* > 0.1) to funnel plots, was utilized to distinguish detail reasons of publication bias. Results of SSI (Figure 7A) indicated many studies were in areas of none-statistical significance (*p* > 0.1), which suggested that the origin of asymmetry may be more likely due to publication bias. Furthermore, results of IgA (Figure 7B), IgM (Figure 7C), IgG (Figure 7D), CD4+ (Figure 7E), CD8+ (Figure 7F), and CD4+/CD8+ (Figure 7G) presented that a great majority of missing studies were in areas of higher statistical significance (*p* < 0.01), which indicated the origin of asymmetry was most likely to be due to undetected factors rather than publication bias. Subsequently, we traced the original researches again, speculating that studies with a small sample size, ITT analysis, and missing blinding in many studies may account for those undetected bias. These factors may influence our conclusions potentially.

#### Metaregression Analysis

**Figure 8:**
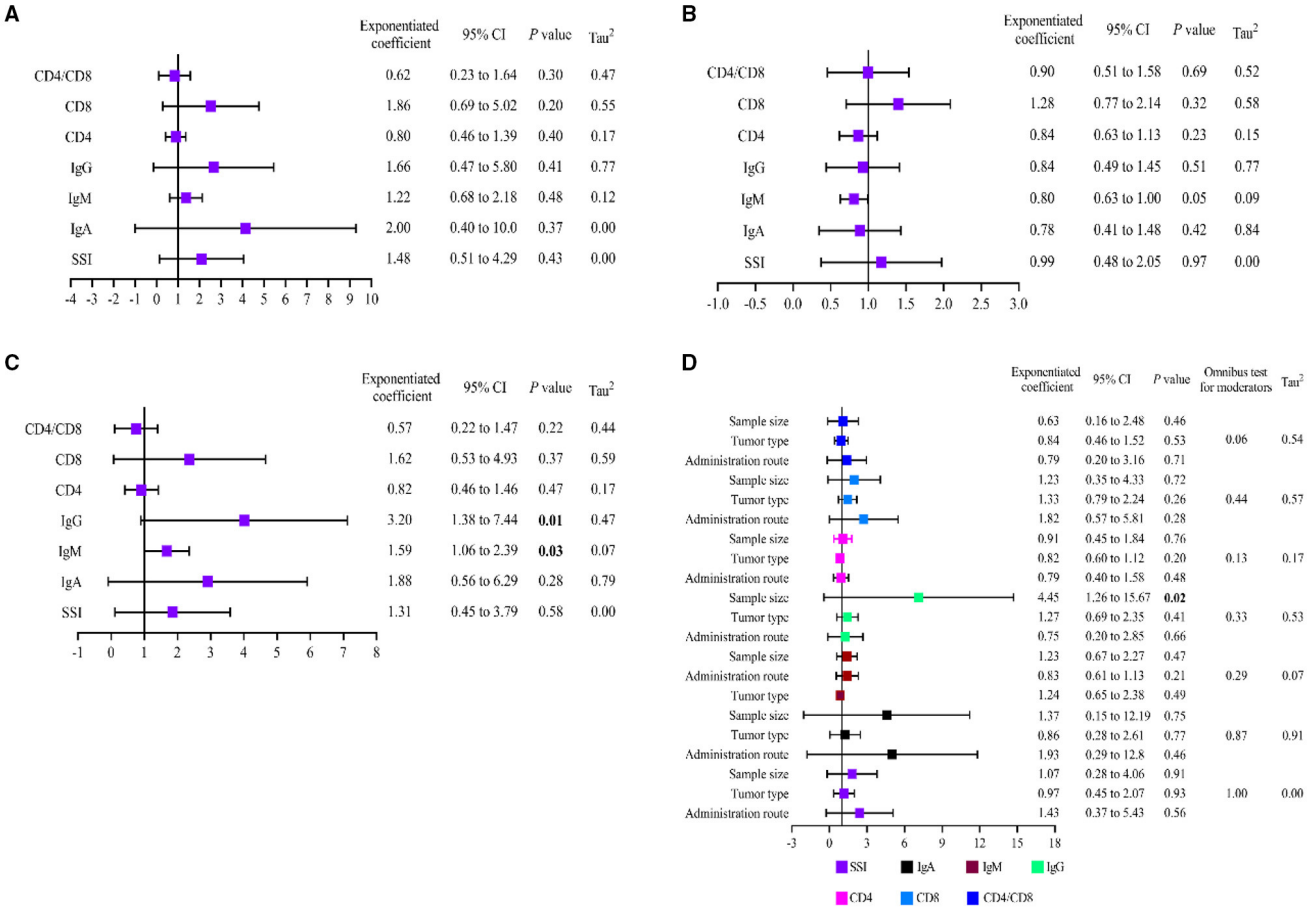
Results of metaregression analysis. **(A)** Univariate analysis of administration route; **(B)** Univariate analysis of tumor type; **(C)** Univariate analysis of total sample size; **(D)** Multivariate analysis of all covariates. SSI, surgical site infection.

Metaregression was performed to assess the effect of underlying confounding factors on pooled effect estimation and to seek the sources of heterogeneity. The following covariates were predicted as potential factors premeditatedly: ① Administration route (PN or EN) of glutamine; ② Tumor type (Colon/rectal/CRC); ③ Total sample size (<100 or ≥100). Overall, univariate analysis indicated the administration route (PN or EN) of glutamine (Table 2, Figure 8A) and type of tumor (Table 2, Figure 8B) had no significant influence on the results of SSI, IgA, IgM, IgG, CD4+, CD8+, and CD4+/CD8+ outcomes (*p* > 0.05). The remaining variable of total sample size had significant influences on the pooled effects of IgM (*p* = 0.03, Table 2, Figure 8C) and IgG (*p* = 0.01, Table 2, Figure 8C). Then, multivariate metaregression was utilized to evaluate the impact of multicovariates on the pooled effects. Three mentioned covariates (administration route of glutamine, tumor type, and total sample size) did not affect the pooled effects of SSI, IgA, IgM, CD4+, CD8+, and CD4+/CD8+, and the heterogeneity did not stem from this model (*p* > 0.05, Table 2, Figure 8D). However, multivariate analysis revealed that the endpoint of IgG was influenced by the covariate of total sample size (*P* = 0.02, Table 2, Figure 8D), which indicated the heterogeneity may originate from this covariate.

**Table 2:** Results of Meta-regression analysis.

**Covariates**	**Univariate analysis**				**Multivariate analysis**		
	**Exponentiated coefficient**	**95% CI**	***P***	**Tau^**2**^**	**Exponentiated coefficient**	**95% CI**	***P***
**Administration route (PN/EN)**							
SSI (12 studies)	1.48	0.51 to 4.29	0.43	0.00	1.43	0.37 to 5.43	0.56
IgA (14 studies)	2.00	0.40 to 10.0	0.37	0.82	1.93	0.29 to 12.8	0.46
IgM (17 studies)	1.22	0.68 to 2.18	0.48	0.12	1.24	0.65 to 2.38	0.49
IgG (17 studies)	1.66	0.47 to 5.80	0.41	0.77	0.75	0.20 to 2.85	0.66
CD4^+^ (16 studies)	0.80	0.46 to 1.39	0.40	0.17	0.79	0.40 to 1.58	0.48
CD8^+^ (15 studies)	1.86	0.69 to 5.02	0.20	0.55	1.82	0.57 to 5.81	0.28
CD4^+^/CD8^+^ (13 studies)	0.62	0.23 to 1.64	0.30	0.47	0.79	0.20 to 3.16	0.71
**Tumor type (Colon/rectal/colorectal cancer)**							
SSI (12 studies)	0.99	0.48 to 2.05	0.97	0.00	0.97	0.45 to 2.07	0.93
IgA (14 studies)	0.78	0.41 to 1.48	0.42	0.84	0.86	0.28 to 2.61	0.77
IgM (17 studies)	0.80	0.63 to 1.00	0.05	0.09	0.83	0.61 to 1.13	0.21
IgG (17 studies)	0.84	0.49 to 1.45	0.51	0.77	1.27	0.69 to 2.35	0.41
CD4^+^ (16 studies)	0.84	0.63 to 1.13	0.23	0.15	0.82	0.60 to 1.12	0.20
CD8^+^ (15 studies)	1.28	0.77 to 2.14	0.32	0.58	1.33	0.79 to 2.24	0.26
CD4^+^/CD8^+^ (13 studies)	0.90	0.51 to 1.58	0.69	0.52	0.84	0.46 to 1.52	0.53
**Total sample size (<100/ ≥100)**							
SSI (12 studies)	1.31	0.45 to 3.79	0.58	0.00	1.07	0.28 to 4.06	0.91
IgA (14 studies)	1.88	0.56 to 6.29	0.28	0.79	1.37	0.15 to 12.2	0.75
IgM (17 studies)	1.59	1.06 to 2.39	0.03	0.07	1.23	0.67 to 2.27	0.47
IgG (17 studies)	3.20	1.38 to 7.44	0.01	0.47	4.45	1.26 to 15.7	0.02
CD4^+^ (16 studies)	0.82	0.46 to 1.46	0.47	0.17	0.91	0.45 to 1.84	0.76
CD8^+^ (15 studies)	1.62	0.53 to 4.93	0.37	0.59	1.23	0.35 to 4.33	0.72
CD4^+^/CD8^+^ (13 studies)	0.57	0.22 to 1.47	0.22	0.44	0.63	0.16 to 2.48	0.46

*NA, Not applicable; SSI, surgical site infection. Significant results are in bold and underlined presentation*.

## Discussion

Overall, findings from this study illustrated that immune functions (including humoral immune function and T cell immune function) can be improved significantly with glutamine in sufferers with CRC. Meanwhile, the main post-operative complications also reduced by glutamine in patients with CRC after surgery. The certainty of conclusion from current study is mainly reflected in the following three aspects. First of all, the critical indicators of humoral immune function, including IgA, IgM, IgG, were significantly increased followed by glutamine intervention. The results of integrated analysis revealed that IgA content (SMD = 1.15, 95% CI: 0.72–1.58) was increased significantly in glutamine group compared with the control group. Meanwhile, the indicator of IgM (SMD = 0.68, 95% CI: 0.48–0.89) and IgG were also significantly increased (SMD = 1.10, 95% CI: 0.70–1.50) in glutamine group. These results demonstrated that glutamine was able to improve the humoral immune function effectively for patients with CRC after radical operation. Secondly, results of integrated analysis revealed that glutamine could regulate T cell immune function effectively of CRC patients after radical surgery. On one hand, the content of CD4+ (SMD = 0.76, 95% CI: 0.53–0.99) and index of CD4+/CD8+ (SMD = 0.92, 95% CI: 0.57–1.28) were increased significantly in glutamine group compared with control group. On the other hand, the content of CD8+ was decreased significantly (SMD = −0.50, 95% CI: −0.91 to −0.10) in glutamine group. These results indicated that glutamine could regulate the disordered immune function of T cell. Thirdly, all indicators of post-operative complications were decreased by glutamine in patients with CRC after surgery. Pooled analysis of SSI (RR = 0.48, 95% CI: 0.30–0.75), anastomotic leakage (RR = 0.23, 95% CI: 0.09–0.61), and LOS (SMD = −1.13, 95% CI: −1.68 to −0.58) were decreased significantly in glutamine group compared with control group. All supporting evidence mentioned above demonstrated that glutamine should be applied as an effective immunenutrition therapy in the treatment of CRC patients after radical surgery.

Immunenutrition support for patients who underwent radical surgery for CRC is widely accepted for reducing the incidence and severity of post-operative complications. However, appropriate assessment and application of immunenutrition therapies were largely neglected (58). Until to now, immunenutrition support is generally recommended by the European Society for Clinical Nutrition and Metabolism (ESPEN) for malnourished patients with cancer (59), and it also coincided with the program of enhanced recovery after surgery (ERAS) (60). Glutamine, a substance of immunenutrition, as the major fuel source for macrophages, lymphocytes, and enterocytes, could increase the immune cell responses and decrease inflammations evidently (61, 62). For lymphocytes, glutamine activates the expression of T cell surface markers CD25, CD45RO, and CD71, promotes directly the proliferation of CD3+ (marker for mature lymphocytes) and T regulatory cells (T-reg) (63, 64). Furthermore, glutamine also reduces lymphokine-activated killer cell activity (64, 65). For monocytes and macrophages, glutamine stimulates antigen presentation, increases expression of surface antigens, and improves antioxidant defenses (66, 67). Due to the high rates of glutamine utilization in lymphocytes, macrophages, and neutrophils, the deficiency of glutamine is mostly like to arise immune dysfunction (68, 69). Previous study has indicated that glutamine could promote T cells differentiated into three subsets (Th1, Th17, and Treg). Meanwhile, glutaminase (GLS), which converts glutamine to glutamate, can promote Th17 but constrain Th1 and CTL effector cell differentiation (70). In addition, a clinical trial reported that glutamine and omega-3 fatty acids not only increased the total lymphocyte count, CD4+, CD8+, complement C3, IgG, IgA in all patients, but also decreased C-reactive protein (CRP) and the rates of wound infection (71). Thus, we come to the conclusion that deficiency of glutamine may lead to impaired immune function and ampliative inflammatory responses of CRC patients after radical surgery. On the contrary, glutamine supplementation could improve immune function and decrease complications after radical surgery in CRC patients.

This current work exerts more attention to the clinical benefits of glutamine in CRC patients after radical surgery. However, it is noteworthy that potential limitations of this integrated analysis should be emphasized. Thirty-one included trials were neither single nor double blinding design, which increases the risk of detection bias. Meanwhile, undetected bias predicted by contour-enhanced funnel plot showed studies with a small sample size and missing ITT analysis may account for potential bias. These factors may have a potential impact on final conclusions. Metaregression by univariate and multivariate analysis found sample size included in original studies was a potential covariate causing significant heterogeneity, and deescalating validity of results in this pooled analysis.

All in all, this meta-analysis with 2,201 patients from 31 RCTs provide pivotal evidence that glutamine supplementation could improve immune function and decrease post-operative complications of CRC patients after radical surgery effectively. When accepting the conclusions of this study, the methodological limitations should be noticed at the same time. It is widely recognized that the management CRC in pre- or post-operative stages is very much needed in the participation of multiple disciplinary team (MDT) and requires long-term medication. Thus, increasing RCTs with larger scale and multidimensional efficacy and nutritional status assessment are extensively required to balance the risk-benefit profile of glutamine in the management of CRC.

## Data Availability Statement

The original contributions presented in the study are included in the article/supplementary material, further inquiries can be directed to the corresponding author/s.

## Author Contributions

TY, XY, and YC performed the search and drafted the manuscript. TY and XY performed the data extraction and analyzed the data. YC, TB, and TX designed the study and amended the original draft. GL, SG, and KX equally involved and contributed into the study conduction. All authors contributed to the article and approved the submitted version.

## Funding

This research was financially supported by the Science and Technology Projects of Guizhou Province [Grant No. 7153, Qiankehe LH (2017)].

## Conflict of Interest

The authors declare that the research was conducted in the absence of any commercial or financial relationships that could be construed as a potential conflict of interest.

## Publisher's Note

All claims expressed in this article are solely those of the authors and do not necessarily represent those of their affiliated organizations, or those of the publisher, the editors and the reviewers. Any product that may be evaluated in this article, or claim that may be made by its manufacturer, is not guaranteed or endorsed by the publisher.
